# How we taught medical students in a tertiary hospital during a pandemic

**DOI:** 10.15694/mep.2020.000163.1

**Published:** 2020-08-10

**Authors:** Wai Ching Lee, ChaoYan Dong, Chen Wee Derrick Aw

**Affiliations:** 1 Sengkang General Hospital

**Keywords:** Covid-19, Clinical Education

## Abstract

This article was migrated. The article was marked as recommended.

## Letter

### Introduction

In Singapore, the realities of the Coronavirus 2019 (COVID-19) pandemic were first felt in January 2020, and the National Alert level raised to the second highest on 7th February 2020 ( Gov.sg, 2020). The three medical schools in Singapore immediately terminated all on-site clinical teaching. Medical students were prohibited from entering hospital premises for any duration or nature of attachment. School had to replace clinical teaching with online learning, simulation-based learning, self-directed learning and research projects.

However, what can replace clinical teaching with ‘live patients’ for medical students? Students acquire and formulate illness scripts by closely following the progress of patients, nurture clinical reasoning skills by participating in the management of a plethora of caseload, and master clinical skills by examining and caring for patients. Through interactions with peers, seniors and healthcare workers in a clinical setting, students also learn the shades of professionalism and bioethics, recognise the value of teamwork and inter-professional collaboration, and appreciate the prevalence and performance of systems-based practice ( Rajasoorya, 2016). Role-modelling and physical immersion within a clinical setting are vital enablers. These skills are required for medical students to become a competent doctor.

Epidemiological predictions speculate that COVID-19 would be in circulation till the end of 2020, at least. Bed occupancy rates in all the hospitals in Singapore have stayed consistently above 90% and more wards had to be converted or opened for COVID patients. Clinician-educators are faced with unprecedented demands with increasing clinical service and teaching responsibilities.

After numerous meetings with the various stakeholders, Singapore Ministry of Health embarked on a phased approach to resume clinical training for medical students. In this light, Sengkang General Hospital (SKH), the largest (1000-bed) regional hospital in Singapore welcomed its first group of 6 medical students on 13 ^th^ April 2020. We wish to share our experiences, considerations and insights from this student attachment.

### Safety was priority

Singapore Ministry of Health placed the onus of preparatory infection control training on the schools. Training on hand hygiene, N95 mask fitting and personal protective equipment (PPE) donning and doffing were conducted in the school one week before commencement of clinical posting. Students also reviewed COVID-19 related education materials prepared by the medical school, as well as case definitions of COVID-19 patients and updated guidelines from the Ministry of Health.

In the hospital, we set a very high expectation for safety compliance amongst healthcare workers and extended these expectations to students. Throughout the clinical attachment, the Dean, the hospital’s Education Office, the clinical education leads and ward supervisors repeatedly highlighted the importance of safety measures to students.

To minimize exposure and cross-infection risks, each student was assigned to a ward or an outpatient team with direct supervision; no cross-team or cross-ward movements were allowed. They were barred from high-risk areas, namely Intensive Care Unit and high-dependency units, isolation/ pneumonia wards, operating theatres, endoscopy centre and the emergency department. Even the medical student lounge was closed till further notice. Students were also prohibited to observe and perform aerosol-generating procedures. They were required to always wear at least a surgical mask and adhere to social distancing measures within and outside the hospital. Their clinical exposure time was limited to ‘office hours’, with reduced ‘night and weekend duty’ sessions. Overseas travel for emergency purposes was subjected to approval from the school.

For the ease of contact tracing, all students kept an electronic record of the patients they were in physical proximity throughout the day. They reported their temperatures twice daily in two online platforms maintained by the school and the hospital. Students who missed one temperature report would receive a call directly from the education director. All students downloaded the “TraceTogether” app in their mobile devices ( Tang and Mahmud, 2020), a Ministry of Health initiative for epidemiological tracing during epidemics. Students always had to remain contactable by the mobile phone. Those developing fever and/or any respiratory symptoms regardless of travel and/or contact history were to seek treatment at the Staff Clinic during office hours, or emergency department after office hours.

### Dedicated ward/team embedment

Each student was assigned to one inpatient ‘clean team’. These teams managed patients with low risk of COVID-19 and non-pneumonia cases in accordance to Ministry of Health guidelines. The students were placed under the supervision of a dedicated ward consultant to provide an ‘apprenticeship style teaching’ ( Dornan, 2005). They were expected to take ownership of some of the team’s patients by clerking them thoroughly, examining and presenting them to the consultant. The consultant provided feedback and assessment of their skills performances, and then conducted case discussions and recommended areas for self-directed learning. At other times, the other team doctors guided the student on case management (including interactions with patients, caregivers and other healthcare personnel) and supervised their procedures. The generalist model in SKH meant that our students encountered a wide range of general medical cases without needing to cross over to other wards or teams, and that our teachers were capable to teach a wide spectrum of clinical material. Students who compared their experiences with their peers in tertiary institutions commented that their experience here stood out as enriching in these times of constraint.

### Focused outpatient clinics

With the exception of high-volume and/or low-manpower specialties (dermatology, haematology, gastroenterology), outpatient specialist clinics were restructured to allocate resources to the COVID frontline. All patients were temperature-screened from the hospital lobby, and symptom-screened at the outpatient reception. Students were guided by the clinic consultant through the “apprenticeship” method as per inpatient setting. Assessments such as Mini-CEX or case-based discussion were done in a timely manner as per normal context. Students assigned to the outpatient clinics were not be allowed to enter the inpatient wards in accordance to team segregation protocols, but rotated through different clinics.

### Supplementary bedside group teaching

As bedside teaching in groups were suspended, we plan to use secured video-conferencing apps for students to perform clinical interview and physical examination skills with a clinical teacher by the bedside or with another partner (if in outpatient setting). Their peers will witness the patient encounter on their mobile devices. This will be done with the patients’ consent; no recordings will be allowed. The teacher will then proceed to engage students with case-based discussion, feedback and assessment. We envisage this process to be challenging for the tutors, so a learning curve is expected. We intend to conduct in-house “training modules” to provide guidance to clinical teachers. Finally, we have had significant success with interactive online teaching using Microsoft Teams and Zoom, and plan to continue using these software to conduct additional tutorials for students.

### Conclusion

The raging COVID-19 pandemic has proved to be an unprecedented time for medical educators worldwide. Teachers and students were under immense pressure to juggle multiple responsibilities in an unpredictable climate, and the stakes are high. The wheels of paradigm change in clinical teaching have started running, and we must innovate and adjust so as to continue providing quality clinical education while maintaining safety as priority. We are gratified to hear that our students felt much more confident to graduate by the end of their posting.

## Notes On Contributors

Dr Wai Ching Lee is a Consultant Internal Medicine in Sengkang General Hospital, Singapore and presently the undergraduate director for the General Medicine Department. Her clinical interest would be in acute medicine, transitional care and metabolic medicine. Her medical education interest would be on curriculum development, faculty development and mentoring. ORCID: https://orcid.org/0000-0002-6597-4426

Dr Chaoyan Dong is a medical educator, working at Sengkang General Hospital, Singapore. She is heavily involved in educational office planning of the hospital. Her main interest includes medical simulation, technology in teaching and faculty development. She has several research in these areas. ORCID: https://orcid.org/0000-0002-9912-0998

Adjunct Associate Professor Chen Wee Derrick Aw is a senior consultant dermatologist and physician in Sengkang General Hospital. His subspecialty interests are acne, psoriasis, eczema and hospital dermatology. He is highly involved in medical education as the Associate Dean of the hospital. ORCID: https://orcid.org/0000-0003-0517-2082
