# Supplementary material for: How we taught medical students in a tertiary hospital during a pandemic
Source: MedEdPublish (2016). 2020 Aug 10;9:163. [Version 1] doi: 10.15694/mep.2020.000163.1 (PMC10702651; doi:10.15694/mep.2020.000163.1)
Supplement: Peer review history

# Reviewer report: Reviewer response for version 1

**DOI:** 10.21956/mep.20105.r30993

**Published:** 2020-11-06

**Authors:** Ken Masters

**Affiliations:** 1 Sultan Qaboos University

This review has been migrated. The reviewer awarded 4 stars out of 5 An interesting short letter on how medical students were taught in a tertiary hospital in Singapore, during the COVID-19 pandemic. The letter shows a very highly-organised approach, involving all levels of management, where the smallest of details were taken into account, including detailed reporting and follow-up. From the description, it appears that the process and activities would be a useful model for many others to follow. It would be interesting to see how adaptations were made as the pandemic progressed. I hope to see some detailed research papers based on these activities in the future.

Reviewer Expertise:

NA

No decision status is available

# Reviewer report: Reviewer response for version 1

**DOI:** 10.21956/mep.20105.r30991

**Published:** 2020-08-18

**Authors:** P Ravi Shankar

**Affiliations:** 1 American International Medical University

This review has been migrated. The reviewer awarded 4 stars out of 5 This is an important manuscript considering that many medical schools are considering restarting a certain amount of clinical teaching for their students. We had a number of articles from Singapore about teaching-learning during the pandemic. The authors started with a batch of six students in April. How many students are being trained right now? What are the challenges involved in providing clinical training to a larger number of students?A major challenge for many medical schools will be the large undergraduate student intake. When a class consists of 250 students then ensuring clinical posting while at the same time focusing on personal protection and social distancing will be challenging.

Reviewer Expertise:

NA

No decision status is available

# Reviewer report: Reviewer response for version 1

**DOI:** 10.21956/mep.20105.r30992

**Published:** 2020-08-10

**Authors:** Hebat Allah A. Amin

**Affiliations:** 1 Department of Pathology, Helwan University, Faculty of Medicine

This review has been migrated. The reviewer awarded 4 stars out of 5 It's a very interesting and important topic. Clinical teaching remains a major challenge in the Pandemic Era. However, we need more detailed case studies to share experiences. I hope you can develop a Step-by-step guide with associated case study of your interesting experience.Thanks for your innovative ideas.

Reviewer Expertise:

NA

No decision status is available
